# Determinants of exposure to acrylamide in European children and adults based on urinary biomarkers: results from the “European Human Biomonitoring Initiative” HBM4EU participating studies

**DOI:** 10.1038/s41598-023-48738-6

**Published:** 2023-12-02

**Authors:** Sandra F. Fernández, Michael Poteser, Eva Govarts, Olga Pardo, Clara Coscollà, Thomas Schettgen, Nina Vogel, Till Weber, Aline Murawski, Marike Kolossa-Gehring, Maria Rüther, Phillipp Schmidt, Sónia Namorado, An Van Nieuwenhuyse, Brice Appenzeller, Kristín Ólafsdóttir, Thorhallur I. Halldorsson, Line S. Haug, Cathrine Thomsen, Fabio Barbone, Marika Mariuz, Valentina Rosolen, Loïc Rambaud, Margaux Riou, Thomas Göen, Stefanie Nübler, Moritz Schäfer, Karin H. A. Zarrabi, Ovnair Sepai, Laura Rodriguez Martin, Greet Schoeters, Liese Gilles, Karin Leander, Hanns Moshammer, Agneta Akesson, Federica Laguzzi

**Affiliations:** 1 Foundation for the Promotion of Health and Biomedical Research in the Valencian Region, FISABIO-Public Health, Av. Catalunya, 21, 46020 Valencia, Spain; 2 Center for Public Health, Department of Environmental Health, Medical University of Vienna, Vienna, Austria; 3 VITO Health, Flemish Institute for Technological Research (VITO), Mol, Belgium; 4 Public Health Directorate of Valencia, Av. Catalunya, 21, 46020 Valencia, Spain; 5 Department of Analytical Chemistry, University of Valencia, Doctor Moliner 50, 46100 Burjassot, Spain; 6 Institute for Occupational, Social and Environmental Medicine, Medical Faculty, RWTH Aachen University, Pauwelsstrasse 30, 52074 Aachen, Germany; 7 German Environment Agency (UBA), Dessau-Roßlau, Berlin, Germany; 8 Department of Epidemiology, National Institute of Health Doutor Ricardo Jorge, Lisbon, Portugal; 9 Comprehensive Health Research Center, Universidade NOVA de Lisboa, Lisbon, Portugal; 10 Public Health Research Centre, NOVA National School of Public Health, Universidade NOVA de Lisboa, Lisbon, Portugal; 11 Laboratoire National de Santé (LNS), 3555 Dudelange, Luxembourg; 12 Human Biomonitoring Research Unit, Department of Precision Health, Luxembourg Institute of Health (LIH), 1 A-B, Rue Thomas Edison, 1445 Strassen, Luxembourg; 13 Department of Pharmacology and Toxicology, University of Iceland, Reykjavík, Iceland; 14 Faculty of Food Science and Nutrition, School of Health Sciences, University of Iceland, Reykjavík, Iceland; 15 Norwegian Institute of Public Health, Lovisenberggata 8, 0456 Oslo, Norway; 16 Department of Medicine, Surgery and Health Sciences, University of Trieste, Ospedale di Cattinara, Strada di Fiume 447, 34149 Trieste, Italy; 17 Central Directorate for Health, Social Policies and Disability, Friuli Venezia Giulia Region, Riva Nazario Sauro, 8, 34124 Trieste, Italy; 18 Santé Publique France, SpFrance, 12, Rue du Val d’Osne, 94415 Saint-Maurice, France; 19 Institute and Outpatient Clinic of Occupational, Social and Environmental Medicine, Friedrich-Alexander-Universität Erlangen-Nürnberg, Henkestraße 9-11, 91054 Erlangen, Germany; 20 UK Health Security Agency, London, SE1 8UG UK; 21 Department of Biomedical Sciences, University of Antwerp, 2610 Antwerp, Belgium; 22 Unit of Cardiovascular and Nutritional Epidemiology, Institute of Environmental Medicine, Karolinska Institutet, Nobels Väg 13, Box 210, 17177 Stockholm, Sweden

**Keywords:** Epidemiology, Predictive markers, Environmental monitoring, Nutrition

## Abstract

Little is known about exposure determinants of acrylamide (AA), a genotoxic food-processing contaminant, in Europe. We assessed determinants of AA exposure, measured by urinary mercapturic acids of AA (AAMA) and glycidamide (GAMA), its main metabolite, in 3157 children/adolescents and 1297 adults in the European Human Biomonitoring Initiative. Harmonized individual-level questionnaires data and quality assured measurements of AAMA and GAMA (urine collection: 2014–2021), the short-term validated biomarkers of AA exposure, were obtained from four studies (Italy, France, Germany, and Norway) in children/adolescents (age range: 3–18 years) and six studies (Portugal, Spain, France, Germany, Luxembourg, and Iceland) in adults (age range: 20–45 years). Multivariable-adjusted pooled quantile regressions were employed to assess median differences (β coefficients) with 95% confidence intervals (95% CI) in AAMA and GAMA (µg/g creatinine) in relation to exposure determinants. Southern European studies had higher AAMA than Northern studies. In children/adolescents, we observed significant lower AA associated with high socioeconomic status (AAMA:β =  − 9.1 µg/g creatinine, 95% CI − 15.8, − 2.4; GAMA: β =  − 3.4 µg/g creatinine, 95% CI − 4.7, − 2.2), living in rural areas (AAMA:β =  − 4.7 µg/g creatinine, 95% CI − 8.6, − 0.8; GAMA:β =  − 1.1 µg/g creatinine, 95% CI − 1.9, − 0.4) and increasing age (AAMA:β =  − 1.9 µg/g creatinine, 95% CI − 2.4, − 1.4; GAMA:β =  − 0.7 µg/g creatinine, 95% CI − 0.8, − 0.6). In adults, higher AAMA was also associated with high consumption of fried potatoes whereas lower AAMA was associated with higher body-mass-index. Based on this large-scale study, several potential determinants of AA exposure were identified in children/adolescents and adults in European countries.

## Introduction

Acrylamide (AA) is a genotoxic food-processing contaminant classified as probably carcinogenic to humans (group 2A) by the International Agency for Research on Cancer (IARC)^1^. It is mainly formed in commonly consumed food, existing in high content in starch e.g., coffee, crisps, fried potatoes, biscuits and cereals, when processed at temperatures above 120 °C under low moisture conditions^2^. AA can be also formed from acrolein, exists in smoking tobacco and, in the occupational setting, it is used as a chemical for the production of polyacrylamides^3^. However, the latter source of AA exposure is considered of less concern^4^.

In vivo and in vitro studies have shown that AA and its main metabolite glycidamide (GA) are carcinogenic, neurotoxic, reprotoxic and toxic for the developmental system^3^. Emerging evidence also links AA exposure to several other diseases^5–8^. In humans, the association between AA and cancer risk, investigated in epidemiological studies, remains unclear^9^. Recently, the European Food Safety Agency (EFSA) reported additional evidence of its genotoxic and non-genotoxic effects and concluded that a risk associated to dietary intake of AA cannot be discarded^10^.

Despite the adoption of mitigation, monitoring and regulatory measures at European level to reduce AA content in food^11,12^, dietary exposure to AA still remains widespread representing a global concern in the general population^3^.

Identification of determinants of exposure might be of great importance as a first step to implement effective measures to reduce AA exposure, especially in vulnerable population groups^13^. Also, it might help to explain the high variability in AA exposure across different populations and countries^14^.

Current knowledge on determinants of AA exposure in the general European population is limited, especially in children^15–17^. Human biomonitoring studies could be useful tools to identify potential factors of exposure to chemical pollutants in children and adults by measuring them and/or their metabolites (biomarkers) in biological samples, such as urine, blood, hair, together with the use of questionnaires^18,19^. For AA, the validated biomarkers are AA and its metabolite GA measured as hemoglobin adducts in blood, including cord blood^20^, and as mercapturic acids in urine samples. These biomarkers are used as long- and short-term exposure biomarkers to this compound, respectively^14^. In addition, evidence suggests that acrylamide levels could also be measured in other human samples, such as breast milk and placenta^21^.

Hence, we aim to assess potential determinants of exposure to AA measured via its urinary biomarkers, AAMA (N-acetyl-S-(2-carbamoylethyl)-L-cysteine) and GAMA (N-acetyl-S-(2-carbamoyl-2-hydroxyethyl)-L-cysteine), in children/adolescents and adults using harmonized data from the European Human Biomonitoring Initiative HBM4EU participating studies, covering different European regions.

## Materials and methods

### Study design, data sources and data collection

The present study was based on the participating studies in the HBM4EU initiative (https://www.hbm4eu.eu/), where newly harmonized, individual-level data, were produced within the so-called HBM4EU Aligned Studies sampled between 2014 and 2021. The HBM4EU survey leveraged existing European capacity by incorporating both new/ongoing and recently conducted studies establishing the first large-scale joint effort to align and harmonize ongoing European HBM initiatives. Key factors contributing to the alignment of these studies include target populations, biomarker analysis quality assurance/control program, data handling, and statistical procedures, all implemented through standardized protocols^22–24^. This unique material enhances inter-study/country comparability. Despite efforts in aligning and harmonizing data, variations persisted in certain variables, such as time sampling (confined to the period 2014–2020), biological matrices, and questionnaires. These differences were acknowledged, and strategies were employed to minimize them, including the use of standardized variables retrieved by the questionnaire and conversion factors for different matrices^22,25^. The complete sampling scheme for the inclusion, combination and data harmonization of HBM4EU Aligned Studies is fully described in Gilles et al.^22^. Two additional HBM studies which generated individual-level data on urinary AA biomarkers outside the HBM4EU initiative were included and harmonized following the same procedure. That is, the German Environmental Survey, 2014–2017, (GerES V)^17^ on children/adolescents (3–18 years old, only a subset there of being part of the Aligned Studies), and the BETTERMILK study (2015)^26^ on adult women (20–45 years old).

The criteria for inclusion of studies were: samples in European children/adolescents (3–18 years old) or adults (20–45 years old), in which AA biomarkers were measured in urines collected between 2014 and 2021, and providing questionnaire data to be used for the investigation of exposure determinants.

The full description of data access permissions and ethical considerations has been described elsewhere^22^. In brief, all the participating studies followed methods in accordance with national and European guidelines and ethics regulation. Each of the country's Ethics Committees approved the study (The Regional Committees for Medical and Health Research Ethics in Norway; the Ethics Committees of the University of Udine and the Institute for Maternal and Child Health—IRCCS Burlo Garofolo, Trieste, Italy; Ile-de-France Protection, The French Data Protection Agency, French Advisory Committee on Information Processing for Research and The French National Agency for Medicines and Health Products' Safety, France; the Ethics Commission of the Berlin Chamber of Physicians, the Medical Association Westfalen-Lippe, the Medical Faculty of the University of Münster and Medical Association of the Saarland and the Federal Officer for Data Protection and Freedom of Information, Germany; The National Bioethics Committee, Iceland; the National Ethical committee of Luxembourg, Luxembourg; the Ethical Committees of the National Institute of Health Doutor Ricardo Jorge, the Regional Health Administrations of North, Center, Lisbon, Tagus Valey, Alentejo, Algarve, the Health Service of the Autonomous Region of Madeira and of the Hospital of Horta, Portugal; the Clinical Research Ethics Committee of the Public Health Directorate, the Center for Public Health Research of the Valencian Government, and the Biomedical Scientific Ethic Committee of the University and Polytechnic Hospital “La Fe”, Spain). Written informed consent was obtained from all participants. For children, the written consent was obtained by legal tutors. In general, it was the child's parents who signed the consent. However, there may be differences in the exact procedure between the participating studies i.e. in some countries approval of one parent was sufficient whereas in other countries consent from both parents may be needed^22^. Each study also confirmed that informed consent and approval were in place for secondary use of the collected data.

In total, 4 studies for children/adolescents (n = 3157) and 6 studies for adults (n = 1297) were included.

### Determinants of exposure

Determinants of exposure were selected based on either prior knowledge (e.g., smoking, BMI, and dietary factors) and/or a non-hypothesis-based approach. The selection was limited due to the availability of data in each participating study since questionnaires differed between some surveys. A full description of the variables considered in this study, together with the harmonization, as well as the treatment as continuous or categorical variables in the association with AA urinary biomarkers, is presented in Supplementary Table S1.

### Urinary levels of AA biomarkers: chemical analysis

Urine sampling strategies across studies were either 24-h (n = 1 study for adults), first-morning spot (n = 2 studies for children/adolescents and 3 studies for adults) or random spot (n = 2 studies for children/adolescents and 2 studies for adults) urine samples. The individual urinary concentrations of AAMA and GAMA obtained from each study were generated using different analytical methods, but their comparability was generally guaranteed by the HBM4EU quality assurance/quality control (QA/QC) programme (further details in Supplementary Information SI-1). The AAMA and GAMA urinary levels were standardized for creatinine (µg/g creatinine) for children/adolescents and adults, to account for variation in dilution and to increase comparability of the data.

### Statistical analysis

Descriptive summary statistics of AAMA and GAMA urinary levels (mean, standard deviation, 10th, 25th, 50th, 75th and 90th percentiles) were calculated for children/adolescents and adults, and by European geographical region if available. As quantification frequencies (QFs) of AAMA and GAMA in urine were > 92% in all the studies, those concentration values reported as < LoQ were excluded from the analysis (n = 2 for AAMA, and n = 2 for GAMA in children; n = 14 for AAMA, and n = 17 for GAMA in adults). This decision was supported by experts in the field and statisticians of the HBM4EU, concluding that this exclusion ensured an improvement of homogeneity and accuracy of the data for analysis. Median regression models were employed to investigate the association between continuous urinary levels of AAMA and GAMA and potential exposure determinants in children/adolescents and adults. This statistical approach allows to regress any percentile of the outcome distribution. As the urinary biomarkers of AA tend to have a skewed distribution, the median regression might be considered a better summary measure than the mean and thus preferable to the classical linear regression. Since results from the 10th, 25th, 50th, 75th, and 90th percentile did not show different results across the percentiles of the distribution, we decided to present only those based on the median regression (50th percentile). The results, expressed as beta coefficients and 95% confidence intervals (95% CI), need to be interpreted as median differences in AAMA and/or GAMA in relation to the corresponding predictor, adjusted for all other factors included in the model. To account for potential heterogeneity among studies, we included in the regression model a study-specific fixed effect. The selection of determinants to be included in the multiple regression models was based on data availability, prior findings, knowledge of the field, and collinearity. To retain statistical power, missing values in categorical predictors were treated as a separate category (known as missing indicator method or “dummy variable adjustment”). Because cigarette smoking is known to increase the levels of AA^3^ by 3–4 times^27^, main analyses were presented excluding active smokers (n = 39 in children/adolescents, and n = 238 in adults).

Additional analyses were performed including both smokers and non-smokers and by participating studies.

STATA software (STATA version 12.1, Corp, College Station, TX, USA) was used to perform all statistical analyses.

## Results

The HBM studies providing data on individual AAMA and GAMA levels in urine of European children/adolescents (median age: 10, IQR: 7–13 years old) and adults (median age: 33, IQR: 28–37 years old) are summarized in Table 1. In total, urinary data on AA metabolites of 3157 children/adolescents and 1297 adults were collected from different countries including European studies from Northern (Norway and Iceland), Southern (Italy, Spain and Portugal) and Western Europe (France, Germany and Luxembourg). No studies on AA urinary levels from Eastern European countries were available and/or agreed to participate. Out of the total number of children/adolescents, 81% were based on studies from Western countries (France and Germany), 9.5% from the North (Norway), and 9.5% from the South (Italy). In adults, the participating studies were more equally distributed within the European regions, with 53% of the individuals being from the West (France, Germany, Luxembourg), 31% from the South (Spain and Portugal), and 16% from the North (Iceland). Sampling years ranged from 2014 to 2017 in studies on children/adolescents, and from 2014 to 2021 in those on adults. Distribution of exposure determinants by geographical area in children and adults, respectively, are shown in Supplementary Table S2.

**Table 1:** Characteristics of the HBM4EU participating studies with urinary levels of AA in children/adolescents and adults.

Population group (total number) median age (IQR, years)	Geographical region	Country	Study acronym^a^	Age years median (IQR)	Sampling year	Type of urine sample for AA analysis	N	HBM4EU QA/QC label^b^
Children/adolescents (3158*) 10 (7–13)	North	Norway	NEBII-NO	10 (9–11)	2016–2017	Random spot urine	299	A
	South	Italy	NACII-IT	7 (7–7)	2014–2016	Random spot urine	300	A
	West	France	ESTEBAN-FR	9 (7–10)	2014–2016	First-morning spot urine	300*	A
		Germany	GerESV-DE	11 (7–14)	2015–2017	First-morning spot urine^c^	2,259	B
Adults (1298**) 33 (28–37)	North	Iceland	Diet_HBM-IS	31 (26–35)	2019–2021	Random spot urine	203**	A
	South	Spain	BETTERMILK	33.5 (31–37)	2015	First-morning spot urine	117	C
		Portugal	INSEF-ExpoQuim-PT	35 (32–37)	2019–2020	First-morning spot urine	294**	A
	West	France	ESTEBAN-FR	34 (29–37)	2014–2016	First-morning spot urine	300	A
		Germany	ESB-DE	23 (21–24.5)	2015, 2019, 2021	24-h urine	180	A
		Luxembourg	Oriscav-Lux2-LU	34 (31–37)	2016–2018	Random spot urine	204	A

IQR: Interquartile range; AA: acrylamide.

*One sample was removed due to missing creatinine value in the ESTEBAN study (299 samples with creatinine value), so finally 3157 urine samples of children/adolescents were included in the present research.

**There were 17 urine samples with missing values for GAMA, and one urine sample with missing values for AAMA, 3 of them from INSEF and 14 from Diet_HBM, therefore, a total number of 1297 and 1281 urine samples of adults were considered for AAMA and GAMA analysis, respectively.

^a^NEBII-NO: “the Norwegian Environmental Biobank II”, Norway (Norwegian Institute of Public Health, NIPH); NACII-IT: "the Northern Adriatic cohort II", Italy (Section of Hygiene and Epidemiology within the Department of Medical and Biological Sciences of the University of Udine, EPIUD); ESTEBAN-FR: "the *Etude de santé sur l’environnement, la biosurveillance, l’activité physique et la nutrition"*, France (Agence Nationale De Sante Publique, ANSP); GerESV-DE: "the German Environmental Survey 2014–2017", Germany (German Environment Agency, UBA); Diet_HBM-IS: "the Icelandic National Dietary Survey", Iceland (University of Iceland, UI); BETTERMILK: "Exposure to persistent and emerging contaminants in infants through breast milk. Characterization of associated environmental and dietary factors and risk assessment, Spain (Foundation for the Promotion of Health and Biomedical Research of the Valencian Region, FISABIO); INSEF-ExpoQuim-PT: the "Exposure of the Portuguese Population to Environmental Chemicals: a study nested in the National Health Examination Survey", Portugal (National Institute of Health Dr. Ricardo Jorge, INSA); ESB-DE: "the Environmental Specimen Bank", Germany (UBA); Oriscav-Lux2-LU: "the Observation of cardiovascular risk factors in Luxembourg", Luxembourg (Laboratoire national de santé, LNS).

^b^Labels of the HBM4EU QA/QC programme: Label A = "Biomarker data quality assured by HBM4EU QA/QC programme"; Label B = "Biomarker data generated before HBM4EU QA/QC programme but deemed comparable by HBM4EU Quality Assurance Unit (QAU)"; Label C = "Biomarker data generated before HBM4EU QA/QC programme but comparability not guaranteed by HBM4EU Quality Assurance Unit (QAU)" (see further details in Supplementary SI-1).

^c^Some samples were defined as random spot urines, since they differed from the standard definition of first-morning urine sample provided in the HBM4EU protocol (e.g. sampled too early in the morning or too late).

Descriptive data on urinary AAMA and GAMA in µg/g creatinine in children/adolescents and adults, and by participating studies are summarized in Table 2. Quantifiable levels of AA metabolites were identified in almost all urine samples, with quantification frequencies ranging from 99 to 100% in children/adolescents, and from 92 to 100% in adults for AAMA and GAMA, respectively. AAMA and GAMA concentrations were fairly similar in children/adolescents and adults, but with a slight tendency of being higher in the youngest age group (Table 2).

**Table 2:** AAMA and GAMA urinary levels (µg/g creatinine) in European children/adolescents and adults (including smokers), by European geographical regions and by participating studies.

Population group	Metabolite	European region	Study acronym	N samples	LOQ	N samples > LOQ	QF (%)	Mean (SD)	P10	P25	P50	P75	P90
Children/adolescents	AAMA	South	NACII-IT	300	3.2	298	99.3	100.2 (94.8)	37.8	55.7	78.7	119.4	181.0
		West	ESTEBAN-FR	299	3.2	299	100	110.2 (114.9)	42.0	57.1	83.9	126.5	193.0
			GerESV-DE	2259	1	2259	100	74.6 (72.7)	30.1	40.1	57.3	84.8	128.6
			Total	2558	–	2558	100	78.8 (79.6)	30.6	41.8	59.9	89.8	137.7
		North	NEBII-NO	299	1	299	100	62.9 (56.4)	27.6	34.5	51.4	75.1	102.4
		Total	All regions	3157	–	3155	99.9	79.3 (79.7)	30.4	41.9	60.7	90.8	139.2
	GAMA	South	NACII-IT	300	1	298	99.3	34.5 (19.9)	16.6	22.4	30.7	40.0	55.4
		West	ESTEBAN-FR	299	1	299	100	14.6 (10.2)	6.8	9.0	11.9	17.7	23.1
			GerESV-DE	2259	1	2257	99.9	14.7 (10.6)	6.5	8.7	12.3	17.6	25.0
			Total	2558	–	2556	99.9	14.7 (10.6)	6.6	8.7	12.2	17.6	24.9
		North	NEBII-NO	299	1	299	100	9.5 (5.3)	5.2	6.6	8.3	10.9	14.2
		Total	All regions	3157	–	3153	100	16.1 (13.0)	6.5	8.6	12.5	19.2	29.3
Adults	AAMA	South	INSEF-ExpoQuim-PT	294	3.2	294	100	94.0 (80.8)	35.1	47.1	72.1	111.8	182.9
			BETTERMILK	117	0.25	117	100	89.8 (76.2)	33.2	46.6	70.3	113.3	161.5
			Total	411	–	411	100	92.8 (79.4)	34.9	46.7	70.5	112.5	170.9
		West	ESTEBAN-FR	300	3.2	300	100	162.5 (211.3)	39.5	54.9	91.7	195.7	342.6
			ESB-DE	180	10	166	92	39.9 (25.8)	19.3	25.7	34.6	48.4	69.5
			Oriscav-Lux2-LU	204	1	204	100	42.9 (45.7)	16.1	20.6	28.8	46.2	88.3
			Total	684	–	670	98	94.6 (154.8)	19.8	28.9	48.3	94.3	211.5
		North	Diet_HBM-IS	202	2	202	100	63.8 (59.7)	24.4	33.0	47.3	74.7	105.5
		Total	All regions	1297	–	1283	99	89.2 (123.7)	22.7	34.3	55.1	95.6	188.3
	GAMA	South	INSEF-ExpoQuim-PT	291	1	291	100	28.1 (15.5)	14.8	18.8	25.0	32.9	42.3
			BETTERMILK	117	0.25	117	100	17.2 (10.2)	8.1	10.4	14.3	22.1	29.5
			Total	408	–	408	100	25.0 (15.0)	10.6	15.3	22.4	30.2	40.7
		West	ESTEBAN-FR	300	1	299	99.7	17.3 (21.3)	5.2	7.2	11.2	20.8	34.3
			ESB-DE	180	1	179	99.4	7.6 (3.3)	4.3	5.5	7.1	8.9	11.4
			Oriscav-Lux2-LU	204	1	204	100	8.0 (6.2)	3.7	4.7	6.0	8.2	14.9
			Total	684	–	682	99.7	12.0 (15.3)	4.3	5.6	7.8	12.8	22.3
		North	Diet_HBM-IS	189	3	174	92	8.4 (6.8)	2.2	4.9	7.5	10.3	15.5
		Total	All regions	1281	–	1264	97.9	15.6 (15.7)	4.5	6.5	10.4	21.0	31.2

LOQ: Limit of quantification; QF: quantification frequency (% samples > LOQ); SD: standard deviation; P: percentile.

AAMA and GAMA levels varied by region and were mainly highest in the Southern region compared to Western and Northern countries. The pooled crude and multivariable adjusted median differences in AAMA and GAMA by geographical region for non-smoking children/adolescents and adults are shown in Table 3 (including smokers in Supplementary Table S3). The multi-adjusted results were produced in a model in which the fixed effect of participating studies was omitted due to collinearity with the indicators of geographical area. Higher median AAMA and GAMA levels in both age groups were observed in the Southern region as compared to those observed in Northern Europe. Similar findings were observed after multivariable adjustment for several dietary and non-dietary factors. Higher levels of AAMA and GAMA were also noted in the studies on children/adolescents from the Western region (vs North), even after multivariable adjustment. By contrast, lower levels of AAMA and GAMA were observed in adults from the West compared to the Northern region.

**Table 3:** Pooled crude and multivariable-adjusted median differences in AAMA and GAMA urinary levels (beta coefficients with 95% CI) in relation to European geographical regions (North vs. South and West). Results are presented for non-smoking children/adolescents and adults, respectively.

Population group	Model	AA urinary biomarkers	North reference	European geographical region		N
				South β (95% CI) (µg/g creatinine)	West β (95% CI) (µg/g creatinine)	
Children/Adolescents	Crude	AAMA	Ref	27.3	7.9	2895
				(20.3, 34.3)	(2.7, 13.1)	
		GAMA	–	22.4	3.9	2895
				(21.2, 23.7)	(3.9, 4.8)	
	Multivariable-adjusted^a^	AAMA	–	22.9	26.6	2958
				(− 17.5, 63.3)	(3.0, 50.2)	
		GAMA	–	25.2	7.1	2958
				(18.2, 32.1)	(3.1, 11.2)	
Adults	Crude	AAMA	–	13.3	− 6.9	937
				(6.3, 20.4)	(− 13.3, − 0.5)	
		GAMA	–	12.6	− 0.3	925
				(11.7, 13.5)	(− 1.1, 0.6)	
	Multivariable-adjusted^b^	AAMA	–	14.8	− 21.0	877
				(2.4, 27.3)	(− 36.9, − 5.0)	
		GAMA	–	6.3	− 2.0	867
				(4.3, 8.2)	(− 4.4, 0.4)	

^a^Model adjusted for the following variables: sex, age, passive smoking, sampling year, place of residence (cities, town and rural areas), highest educational level in the household, body mass index (BMI), physical activity, frequency of consumption of seafood, fish, meat, cereals, fried potatoes, bread, fast food, sugar drinks and tea/coffee.

^b^Model adjusted for the following variables: sex, sampling year, age, place of residence (cities, town and rural areas), educational level, physical activity, BMI, frequency of consumption of seafood, fried potatoes, fruits/vegetables, coffee and alcohol.

**Figure 1:**
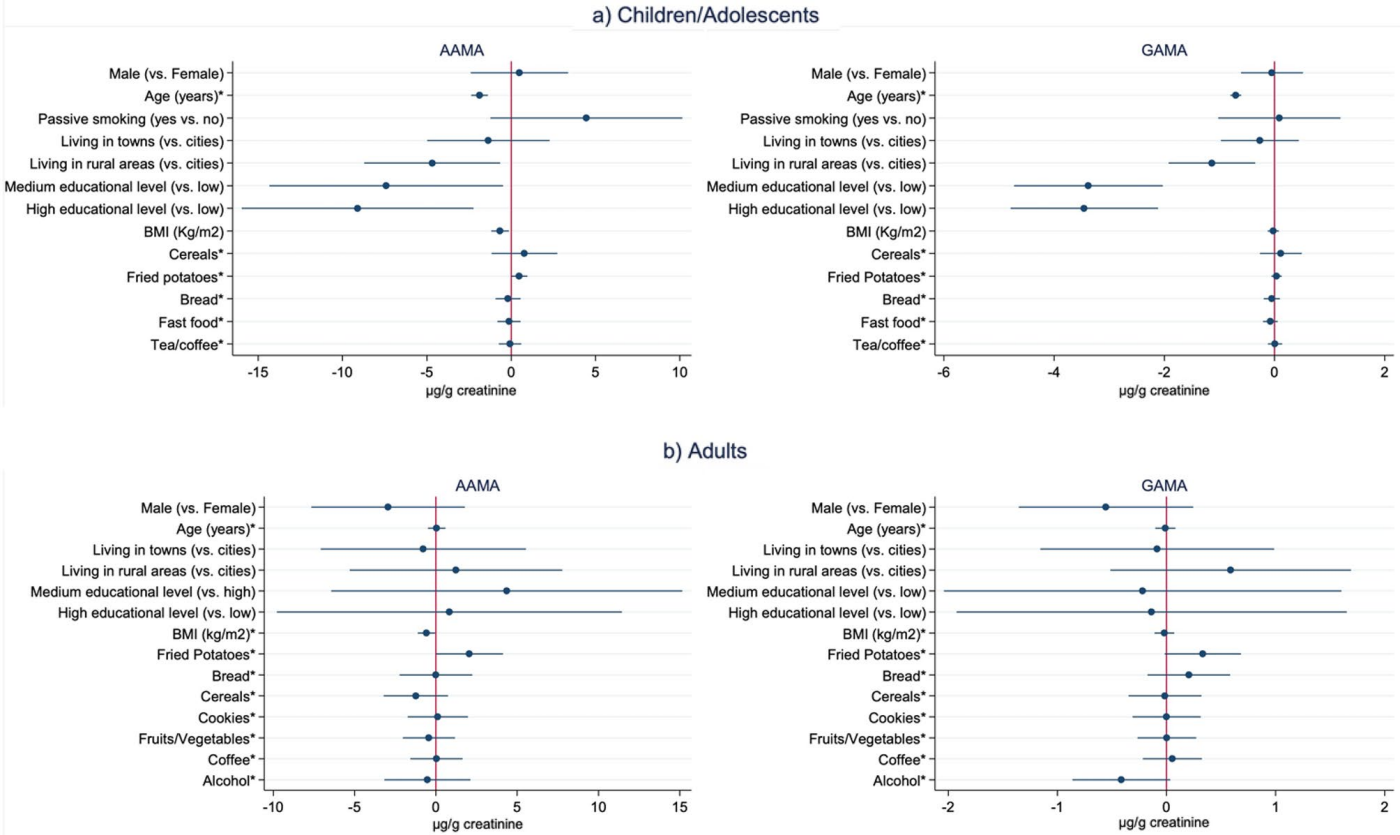
Pooled multivariable-adjusted^1^ median differences in AAMA and GAMA urinary levels (beta coefficients and 95% CI in µg/g creatinine) in relation to non-dietary and dietary determinants. Results are presented for non-smoking children/adolescents (**a**) and adults (**b**), respectively^2^. Educational level classified according to ISCED: international standard classification for education (for children/adolescents referred to the maximum reported in the household); BMI: body mass index. ^1^In children/adolescents, adjustments were made for variables shown in Figure (a) plus participating study, sampling year, physical activity, and consumption of seafood, fish, meat and sugar drinks; in adults, adjustments were made for variables shown in Figure (b) plus participating study, sampling year, physical activity, vegetarian diet, and consumption of seafood, fish, meat and milk. ^2^For a better readability of the results, raw data of the following variables were multiplied by a factor of 25: cereals, fried potatoes, bread and fast food in children/adolescents; and cereals, fruit/vegetables and coffee in adults. *Age, BMI and dietary variables (frequency of consumption) treated as continuous (unit increase).

Results from multivariable-adjusted pooled median regression analysis considering both dietary and non-dietary factors (but not region), excluding smokers in children/adolescents and adults are shown in Fig. 1. In both children/adolescents and adults, lower levels of AAMA were associated with participants having higher BMI (*β* =  − 0.7, 95% CI − 1.2, − 0.2 µg/g creatinine; and *β* =  − 0.6, 95% CI − 1.1, − 0.1 µg/g creatinine, respectively). For children/adolescents, lower levels of AAMA and GAMA were observed in relation to increasing age (*β* =  − 1.9, 95% CI − 2.4, − 1.4 µg/g creatinine; and *β* =  − 0.7, 95% CI − 0.8, − 0.6 µg/g creatinine, respectively), higher education (*β* =  − 9.1, 95% CI − 15.8, − 2.4 µg/g creatinine; and *β* =  − 3.4, 95% CI − 4.7, − 2.2 µg/g creatinine, respectively) and living in rural areas (*β* =  − 4.7, 95% CI − 8.6, − 0.8 µg/g creatinine; and *β* =  − 1.1, 95% CI − 1.9, − 0.4 µg/g creatinine, respectively). In children/adolescents, none of the dietary factors were associated with levels of AAMA and/or GAMA, whereas in adults, higher levels of AAMA were observed in participants that reported a high consumption of fried potatoes (*β* = 2.1, 95% CI 0.2, 4.0 µg/g creatinine).

Results from multivariable-adjusted analysis including smoking children/adolescents and adults are shown in Supplementary Fig. S1. These results were, in general, observed to be in the same direction as the results obtained for the non-smokers.

Results from additional analyses investigating crude and multivariable-adjusted associations between each of the considered determinants and levels of AAMA and GAMA by participating studies (data not shown) pointed out that smoking (active) was clearly associated with higher levels of AAMA and GAMA in all the participating studies when available in the corresponding study dataset.

## Discussion

**Figure 2:**
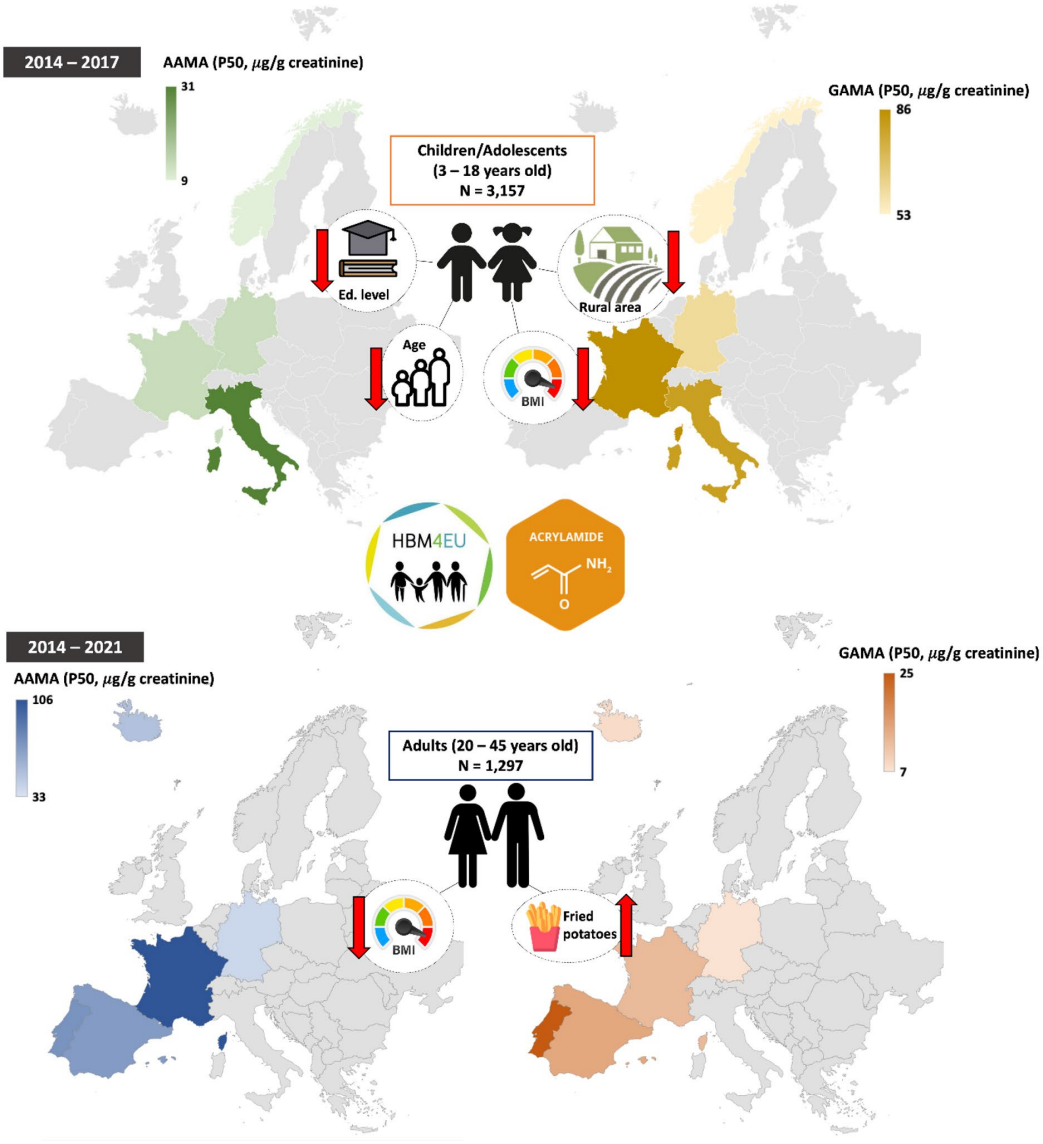
Schematic representation of the results by participating studies (colored countries) in children/adolescents (top) and adults (bottom). The figure depicts the median levels of AAMA (left side) and GAMA (right side) (µg/g creatinine), measured in urine samples collected between 2014 and 2017 in children, and 2014–2021 in adults. Various shades of colors differentiate the levels in both age groups. Exposure determinants associated with AAMA and GAMA levels are depicted on the left and right sides, respectively. Red arrows indicate direction of observed associations with urinary levels of AA biomarkers: an up arrow shows an increase, while a down arrow shows a decrease.

Based on AA data obtained from this large cross-sectional study performed in 3157 children/adolescents from 4 different European countries (Norway, Germany, France and Italy) and 1297 adults from 6 different European countries (Iceland, Germany, France, Luxembourg, Portugal and Spain), several determinants potentially relevant to AA exposure, measured though urinary biomarkers, were identified (Fig. 2).

In particular, for the first time, our study showed that AA exposure, measured through urinary biomarkers, was higher in Southern European countries compared to those from the North, independently of several dietary and non-dietary factors. In comparison, a previous study based on the European Prospective Investigation into Cancer and Nutrition (EPIC) performed in 9 European countries (Sweden, Denmark, France, United Kingdom, The Netherlands, Germany, Spain, Italy, and Greece) including around 30 adults per country (age range 41–60 years old), noted that AA exposure differ by countries, with higher levels in Western countries ^28^, including United Kingdom and The Netherlands, than the remaining countries included in the study. Compared to our data, the EPIC study was performed in a smaller sample size, which may impact the uncertainty of their findings; different European countries were included and haemoglobin adducts of AA in blood were measured. Our observed South-North geographical differences did not seem to be fully explained by dietary and non-dietary determinants investigated here. However, these differences in AA exposure might be still due to unmeasured determinants, i.e., specific regional dietary habits, as well as environmental factors^29^, variability of AA metabolism across populations^30–32^, and the presence of gene-environmental interactions and/or a combination of these^33^.

In children/adolescents, our results of decreasing urinary levels of AA biomarkers in relation to increasing age agree with two previous HBM studies^16,34^ but disagree with most of previous HBM studies^17,35–37^. Also, our findings of decreasing urinary levels of AA in relation to high education level and living in rural areas disagree with earlier HBM studies^16,17,35–37^. These discrepancies might be due to differences between our study and the previous HBM studies in terms of design, study characteristics, and used methodologies, i.e., different urine collection methods, covered age ranges, sampling years and statistical methods. The findings related to age were also confirmed by the higher average levels of AAMA and GAMA in children/adolescents compared to adults observed in our study but also in Poteser et al.^38^. In support of that, European young children have been observed to have higher dietary intake relative to body weight (0.5–1.9 µg/kg bw/day) compared to adolescents and adults (0.4–0.9 µg/kg bw/day), and consume more food containing higher concentrations of AA, such as snacks, biscuits, and cereal-based products, than older population groups^3,29^. In addition, it is worth mentioning that children´s interindividual variation in the activity and polymorphisms of the enzymes involved in the process of AA detoxification has been suggested to be different from adults^39,40^. Regarding the observed association between high urinary AA biomarkers in children/adolescents and low educational level and living in urban areas, some potential explanations can be provided. Firstly, children/adolescents living in a household of low educational level may consume more processed food (more likely to have high levels of AA) than those living in a high educational level environment since they and/or their parents may have little awareness of healthy dietary habits, including cooking methods, which are known to affect AA exposure^41,42^. Secondly, as previously suggested^43^, rural environments are considered greenness locations associated with low exposure to volatile organic compounds present in polluted areas, such as AA. Furthermore, children/adolescents living in urban areas may have easier access to processed and fast food than those living in rural areas^44^, which may easily find local and home-grown food products, such as vegetables and fruits, leading to a lower exposure to AA^45^.

Interestingly, the association between higher BMI and lower AAMA found here for both population groups (children/adolescents and adults) is in line with two previous HBM studies^46,47^ but not with all^16,26,36,37,39,48,49^. The main methodical differences, potentially explaining the divergent results, may be that most of the latter studies were performed in Asian populations which may have different genetics and lifestyle, affecting both BMI, dietary intake and metabolism of AA^50,51^. It has been proposed that body composition can play an important role affecting the metabolism of AA and also its excretion efficiency in humans^48,52,53^. In addition, high BMIs have been associated with increased oxidative stress, deficiency of antioxidant mechanisms and higher cytochrome 2E1 (CYP2E1) activity^54,55^, which may induce lower AAMA and higher production of GA. However, in our study no association between GAMA and BMI was observed.

In general, increased concentrations of urinary biomarkers of AA in relation to higher consumption of fried potatoes in adults confirmed results from most previous HBM studies^16,17,26,36,39,42,46,48,56,57^. The weak or no associations observed between specific dietary determinants and urinary biomarkers of AA, especially in children/adolescents, has previously been reported in several studies^16,17,26,36,56^. One possible explanation for these findings may be that the employment of self-reported questionnaires (as in this study), especially food frequency questionnaires that assess intake of long-term AA exposure (weeks/months/years), may not be captured by urinary AA metabolites, which are known to be short-term biomarkers of dietary AA exposure (half-life ≈ 3.3 h)^39^.

Finally, our results confirmed that smoking is a strong determinant of AA exposure estimated by urinary levels in adults, as previously reported^14,38^. However, it is important to highlight that in our study even though smoking status slightly affected the magnitude of the associations, the overall direction of the associations between urinary AA levels and potential determinants did not. Although the reason is not fully clear, some scientific evidence suggest that smokers may have different activity of the enzymes involved in AA metabolism e.g., CYP2E1^9,58^.

## Strengths and limitations of the study

This study is based on unique data comprising large harmonized high-quality data on urinary levels of AA biomarkers and several exposure determinants from a wide spectrum of European geographical areas. Despite the large effort made to harmonize the data, some heterogeneity across studies could not be avoided, for instance differences in how urine samples were collected and study characteristics. Also, since all the determinants investigated were assessed through self-reported data from questionnaires, bias related to misclassification of exposure determinants cannot be ruled out. Another study limitation is that we might have failed to identify some associations with long-term exposure determinants since urinary AA metabolites are short-term biomarkers. In general, this would most likely cause the estimated coefficients to be biased towards the null due to dilution effects. In addition, these results may not be generalizable to other non- and/or European population groups since the study base of the included HBM4EU participating studies was not representative of the corresponding region/country/European area. Furthermore, the presence of overfitting may have inflated the standard errors making it difficult to achieve statistically significant results.

## Conclusions

In this large high-quality and harmonized data including a wide spectrum of European areas, several potential determinants of AA exposure were identified in both children/adolescents (socioeconomic status, living in rural areas, age, and BMI) and adults (fried potatoes intake and BMI). Our findings provide additional scientific knowledge on exposure determinants of AA, which may form basis to decrease the burden of AA exposure in the European population. Overall, our findings strengthen the evidence that children/adolescents might be a vulnerable group, and, generally, encourage the continuation of monitoring levels of AA biomarkers among the European citizens. In addition, further research is needed to investigate potential determinants, dietary and non-dietary, using well-aligned questionnaires, including genetics and their interactions behind the observed European geographic differences in AA exposure.

### Supplementary Information

Supplementary Information.

## Data Availability

The data that support the findings of this study are available from the Personal Exposure and Health (PEH) Data Platform (https://hbm.vito.be/peh-data-platform) but restrictions apply to the availability of these data, which were used under license for the current study, and so are not publicly available. Data are however available upon reasonable request and with permission of the Data Request Access Committee (DRAC) following the procedures for data access as described here: https://hbm.vito.be/peh-data-platform/data-access-procedure.
