# Publisher Correction: Determinants of exposure to acrylamide in European children and adults based on urinary biomarkers: results from the “European Human Biomonitoring Initiative” HBM4EU participating studies

**DOI:** 10.1038/s41598-024-52775-0

**Published:** 2024-01-29

**Authors:** Sandra F. Fernández, Michael Poteser, Eva Govarts, Olga Pardo, Clara Coscollà, Thomas Schettgen, Nina Vogel, Till Weber, Aline Murawski, Marike Kolossa-Gehring, Maria Rüther, Phillipp Schmidt, Sónia Namorado, An Van Nieuwenhuyse, Brice Appenzeller, Kristín Ólafsdóttir, Thorhallur I. Halldorsson, Line S. Haug, Cathrine Thomsen, Fabio Barbone, Marika Mariuz, Valentina Rosolen, Loïc Rambaud, Margaux Riou, Thomas Göen, Stefanie Nübler, Moritz Schäfer, Karin H. A. Zarrabi, Ovnair Sepai, Laura Rodriguez Martin, Greet Schoeters, Liese Gilles, Karin Leander, Hanns Moshammer, Agneta Akesson, Federica Laguzzi

**Affiliations:** 1 Foundation for the Promotion of Health and Biomedical Research in the Valencian Region, FISABIO-Public Health, Av. Catalunya, 21, 46020 Valencia, Spain; 2 Center for Public Health, Department of Environmental Health, Medical University of Vienna, Vienna, Austria; 3 VITO Health, Flemish Institute for Technological Research (VITO), Mol, Belgium; 4 Public Health Directorate of Valencia, Av. Catalunya, 21, 46020 Valencia, Spain; 5 Department of Analytical Chemistry, University of Valencia, Doctor Moliner 50, 46100 Burjassot, Spain; 6 Institute for Occupational, Social and Environmental Medicine, Medical Faculty, RWTH Aachen University, Pauwelsstrasse 30, 52074 Aachen, Germany; 7 German Environment Agency (UBA), Dessau-Roßlau, Berlin, Germany; 8 Department of Epidemiology, National Institute of Health Doutor Ricardo Jorge, Lisbon, Portugal; 9 Comprehensive Health Research Center, Universidade NOVA de Lisboa, Lisbon, Portugal; 10 Public Health Research Centre, NOVA National School of Public Health, Universidade NOVA de Lisboa, Lisbon, Portugal; 11 Laboratoire National de Santé (LNS), 3555 Dudelange, Luxembourg; 12 Human Biomonitoring Research Unit, Department of Precision Health, Luxembourg Institute of Health (LIH), 1 A-B, Rue Thomas Edison, 1445 Strassen, Luxembourg; 13 Department of Pharmacology and Toxicology, University of Iceland, Reykjavík, Iceland; 14 Faculty of Food Science and Nutrition, School of Health Sciences, University of Iceland, Reykjavík, Iceland; 15 Norwegian Institute of Public Health, Lovisenberggata 8, 0456 Oslo, Norway; 16 Department of Medicine, Surgery and Health Sciences, University of Trieste, Ospedale di Cattinara, Strada di Fiume 447, 34149 Trieste, Italy; 17 Central Directorate for Health, Social Policies and Disability, Friuli Venezia Giulia Region, Riva Nazario Sauro, 8, 34124 Trieste, Italy; 18 Santé Publique France, SpFrance, 12, Rue du Val d’Osne, 94415 Saint-Maurice, France; 19 Institute and Outpatient Clinic of Occupational, Social and Environmental Medicine, Friedrich-Alexander-Universität Erlangen-Nürnberg, Henkestraße 9-11, 91054 Erlangen, Germany; 20 UK Health Security Agency, London, SE1 8UG UK; 21 Department of Biomedical Sciences, University of Antwerp, 2610 Antwerp, Belgium; 22 Unit of Cardiovascular and Nutritional Epidemiology, Institute of Environmental Medicine, Karolinska Institutet, Nobels Väg 13, Box 210, 17177 Stockholm, Sweden

Correction to: *Scientific Reports* 10.1038/s41598-023-48738-6, published online 02 December 2023

The original version of this Article contained an error in the name of the author Federica Laguzzi, which was incorrectly given as Laguzzi Federica.

The original Article has been corrected.

