# Supplementary material for: Determinants of exposure to acrylamide in European children and adults based on urinary biomarkers: results from the “European Human Biomonitoring Initiative” HBM4EU participating studies
Source: Sci Rep. 2023 Dec 2;13:21291. doi: 10.1038/s41598-023-48738-6 (PMC10693547; doi:10.1038/s41598-023-48738-6)
Supplement: Supplementary file 1 — Supplementary Information. [file 41598_2023_48738_MOESM1_ESM.docx]

**SUPPLEMENTARY MATERIAL:**

**Determinants of exposure to acrylamide in European children and adults based on urinary biomarkers: results from the “European Human Biomonitoring Initiative” HBM4EU participating studies**

Sandra F. Fernández^1^, Michael Poteser^2^, Eva Govarts^3^, Olga Pardo^4,5^, Clara Coscollà^1^, Thomas Schettgen^6^, Nina Vogel^7^, Till Weber^7^, Aline Murawski^7^, Marike Kolossa-Gehring^7^, Maria Rüther^7^, Phillipp Schmidt^7^, Sónia Namorado^8,9,10^, An Van Nieuwenhuyse^11^, Brice Appenzeller^12^, Kristín Ólafsdóttir^13^, Thorhallur I. Halldorsson^14^, Line S. Haug^15^, Cathrine Thomsen^15^, Fabio Barbone^16^, Marika Mariuz^17^, Valentina Rosolen^17^, Loïc Rambaud^18^, Margaux Riou^18^, Thomas Göen^19^, Stefanie Nübler^19^, Moritz Schäfer^19^, Karin H. A. Zarrabi^19^, Ovnair Sepai^20^, Laura Rodriguez Martin^3^, Greet Schoeters^3,21^, Liese Gilles^3^, Karin Leander^22^, Hanns Moshammer^2^, Agneta Akesson^22^, and Federica Laguzzi^22*^

*^1^Foundation for the Promotion of Health and Biomedical Research in the Valencian Region, FISABIO-Public Health, Av. Catalunya, 21, 46020, Valencia, Spain*

*^2^Medical University of Vienna, Center for Public Health, Dept. of Environmental Health*

*^3^VITO Health, Flemish Institute for Technological Research (VITO), Mol, Belgium*

*^4^Public Health Directorate of Valencia, Av. Catalunya, 21, 46020, Valencia, Spain*

*^5^Department of Analytical Chemistry, University of Valencia, Doctor Moliner 50, 46100, Burjassot, Spain*

*^6^Institute for Occupational, Social and Environmental Medicine, Medical Faculty, RWTH Aachen University, Pauwelsstrasse 30, D-52074, Aachen, Germany*

*^7^German Environment Agency (UBA), Dessau-Roßlau/Berlin, Germany*

*^8^Department of Epidemiology, National Institute of Health Doutor Ricardo Jorge, Lisbon, Portugal*

*^9^Comprehensive Health Research Center, Universidade NOVA de Lisboa, Lisbon, Portugal*

*^10^Public Health Research Centre, NOVA National School of Public Health, Universidade NOVA de Lisboa, Lisbon, Portugal*

*^11^Laboratoire National de Santé (LNS), 3555 Dudelange, Luxembourg*

*^12^Human Biomonitoring Research Unit, Department of Precision Health, Luxembourg Institute of Health (LIH), 1 A-B, Rue Thomas Edison, L-1445 Strassen, Luxembourg*

*^13^Department of Pharmacology and Toxicology, University of Iceland*

*^14^Faculty of Food Science and Nutrition, School of Health Sciences, University of Iceland*

*^15^Norwegian Institute of Public Health, Lovisenberggata 8, Oslo 0456, Norway*

*^16^Department of Medicine, Surgery and Health Sciences, University of Trieste, Ospedale di Cattinara - Strada di Fiume 447 - 34149 Trieste, Italy*

*^17^Central Directorate for Health, Social Policies and Disability, Friuli Venezia Giulia Region, Riva Nazario Sauro, 8 34124 Trieste, Italy*

*^18^SSanté publique France, SpFrance, 12, rue du Val d'Osne 94 415 Saint-Maurice, France*

*^19^Friedrich-Alexander-Universität Erlangen-Nürnberg, Institute and Outpatient Clinic of Occupational, Social and Environmental Medicine, Henkestraße 9-11, 91054 Erlangen, Germany*

*^20^UK Health Security Agency, London SE1 8UG, UK.*

*^21^Department of Biomedical Sciences, University of Antwerp, 2610 Antwerp, Belgium*

*^22^Unit of Cardiovascular and Nutritional Epidemiology, Institute of Environmental Medicine, Karolinska Institutet, Nobels väg 13, Box 210, 17177, Stockholm, Sweden*

***Corresponding Author:** Laguzzi Federica, Unit of Cardiovascular and Nutritional Epidemiology, Institute of Environmental Medicine, Karolinska Institutet, Nobels väg 13, Box 210, 17177 Stockholm, Sweden. Email: federica.laguzzi@ki.se. Mobile phone: +46 (0)764189125

| **List of Contents** | |
| --- | --- |
| **Table S1** | Detailed information on the variables available in HBM4EU Aligned and participating studies which were included in the main analyses of the association with AAMA and GAMA urinary levels and/or by participating studies: variable name, description of the variable and units/code used for harmonization purposes. |
| **Table S2** | Distribution of selected exposure variables model by geographical area (North, South and West) in children and adults, separately. |
| **Table S3** | Pooled crude and multivariable-adjusted median differences in AAMA and GAMA urinary levels (beta coefficients with 95% CI) in relation to European geographical regions (North vs South and West). Results are presented for the whole sample of children/adolescents and adults, respectively, including passive and active smokers. |
| **Figure S1** | Pooled multivariable-adjusted median differences in AAMA and GAMA urinary levels (beta coefficients and 95% CI in µg/g creatinine) in relation to non-dietary and dietary determinants. Results are presented for the whole sample of children/adolescents (a) and adults (b), respectively, including passive and active smokers. |
| **Supplementary information 1 (SI-1)** | Analytical methods for determination of AAMA and GAMA in urine of the HBM4EU Aligned and participating studies included in the present research. |

**Table S1.** Detailed information on the variables available in HBM4EU Aligned and participating studies which were included in the main analyses of the association with AAMA and GAMA urinary levels and in the additional analyses performed by participating studies: variable name, description of the variable and units/code used for harmonization purposes.

| **Variable name** | **Description of variable** | **Unit/Code** |
| --- | --- | --- |
| **Sample collection** | | |
| study | study name | Studies on children:  2 = NACII-IT  3 = GerESV-DE  4 = NEBII-NO  5 = ESTEBAN-FR  Studies on adults:  23 = ESTEBAN-FR  26 = Diet_HBM-IS  30 = INSEF-ExpoQuim-PT  32 = ESB-DE  33 = Oriscav-Lux2-LU  36 = BETTERMILK |
| matrix | Type of urine sample | US = Urine-spot  UD = Urine-24h  UM = Urine-morning urine |
| samplingyear | year of sample collection | Year |
| samplingmonth | month of sample collection (1 is the first month of the year) | 1 = January  2 = February  3 = March  4 = April  5 = May  6 = June  7 = July  8 = August  9 = September  10 = October  11 = November  12 = December |
| samplingseason | Season of sampling | 1 = spring  2 = summer  3 = autumn  4 = winter |
| **Non-dietary factors** | | |
| age_birth_m^b^ | age of the mother of the participant in years at delivery | number of years |
| ageyears | age in years of the participant at sampling | Indicate number of years |
| birthweight^b^ | birthweight of the participant | grams |
| bmical | body mass index of the participant | kg/m^2^ |
| breastfed^b^ | duration (exclusive and partial) in weeks that participant was breastfed | number of weeks |
| country | country of residence of the participant according to ISO 3166-1 alpha-2 at sampling | Source: http://publications.europa.eu/mdr/resource/authority/country/html/countries-eng.html: Use the 2-letter code (ISO 3166-1 alpha-2). e.g. 'BE' = Belgium |
| degurba | Degree of urbanization of residence at sampling of the participant (place of residence) | 1 = Living in cities 2 = Living in towns/suburbs  3 = Living in rural areas |
| gaweeks^b^ | gestational age in weeks of the participant (child) at birth | number of weeks |
| isced_hh^b^ | Highest education level of the household of the participant (ISCED scale) at sampling | 1 = Low education (ISCED 0-2)  2 = Medium education (ISCED 3-4)  3 = High education (ISCED >=5) |
| isced^a^ | education level of the participant (ISCED scale) at sampling | 1 = Low education (ISCED 0-2)  2 = Medium education (ISCED 3-4)  3 = High education (ISCED >=5) |
| parity_sn^a^ | If participant is female. how many children has she had? | number of children |
| physical_activity | practice of a physical activity by participant | 0 = never  1 = rarely (<1 time/month)  2 = sometimes (<=1 time/month but >= 1 time/month)  3 = often (2-3 times / week)  4 = very often (4-6 times / week)  5 = everyday (> = 7 times / week) |
| physical_activity2 | Has participant completed at least 150 minutes of moderate-intensity aerobic physical activity throughout the week or at least 75 minutes of vigorous-intensity aerobic physical activity throughout the week or an equivalent combination of moderate- and vigorous-intensity activity. | 0 = no  1 = yes |
| region | Geographical region of residence following the United Nations geoscheme for Europe | 1 = North  2 = South  3 = West  4 = East |
| sex | sex of the participant | F = female  M = male |
| smoking | Smoking status of the participant at sampling | 0 = no  1 = yes (current smoker) |
| smoking_passive | Passive smoking exposure at home | 0 = no  1 = yes |
| **Dietary factors** | | |
| alcohol | Alcoholic beverages consumption | 0 = never  1 = occasional (<1/week)  2 = frequent (>=1 week) |
| beer^a^ | beer consumption | 1 = < 1 drink/month  2 = 1-4 drinks/ month 3 = 1-6 drinks/week 4 = 1-3 drinks/per day |
| bread_fd | Frequency of consumption of bread | 0 = never  1 = rarely (<1 time/month)  2 = sometimes (<=1 time/month but >= 1 time/month)  3 = often (2-3 times / week)  4 = very often (4-6 times / week)  5 = everyday (> = 7 times / week) |
| butter_fd^a^ | Frequency of consumption of butter by participant | 0 = never  1 = rarely (<1 time/month)  2 = sometimes (<=1 time/month but >= 1 time/month)  3 = often (2-3 times / week)  4 = very often (4-6 times / week)  5 = everyday (> = 7 times / week) |
| can_drk^a^ | Frequency of consumption of drinks in can by participant | 0 = never  1 = rarely (<1 time/month)  2 = sometimes (<=1 time/month but >= 1 time/month)  3 = often (2-3 times / week)  4 = very often (4-6 times / week)  5 = everyday (> = 7 times / week) |
| can_fd | Frequency of consumption of canned food by participant | 0 = never  1 = rarely (<1 time/month)  2 = sometimes (<=1 time/month but >= 1 time/month)  3 = often (2-3 times / week)  4 = very often (4-6 times / week)  5 = everyday (> = 7 times / week) |
| canteen_fd^b^ | Frequency of consumption of dishes served in the canteen by participant | 0 = never  1 = rarely (<1 time/month)  2 = sometimes (<=1 time/month but >= 1 time/month)  3 = often (2-3 times / week)  4 = very often (4-6 times / week)  5 = everyday (> = 7 times / week) |
| cereal_fd | Frequency of consumption of cereals by participant | 0 = never  1 = rarely (<1 time/month)  2 = sometimes (<=1 time/month but >= 1 time/month)  3 = often (2-3 times / week)  4 = very often (4-6 times / week)  5 = everyday (> = 7 times / week) |
| cheese_fd^a^ | Frequency of consumption of cheese by participant | 0 = never  1 = rarely (<1 time/month)  2 = sometimes (<=1 time/month but >= 1 time/month)  3 = often (2-3 times / week)  4 = very often (4-6 times / week)  5 = everyday (> = 7 times / week) |
| coffee_drk^a^ | Frequency of consumption of coffee | 0 = never  1 = rarely (<1 time/month)  2 = sometimes (<=1 time/month but >= 1 time/month)  3 = often (2-3 times / week)  4 = very often (4-6 times / week)  5 = everyday (> = 7 times / week) |
| cookie_fd^a^ | Frequency of consumption of cookies, biscuits and cakes | 0 = never  1 = rarely (<1 time/month)  2 = sometimes (<=1 time/month but >= 1 time/month)  3 = often (2-3 times / week)  4 = very often (4-6 times / week)  5 = everyday (> = 7 times / week) |
| dietgluten^a^ | Does participant eat gluten free? | 0 = no  1 = yes |
| drink_wtr^a^ | Type of drinking water (most) consumed by participant | 1 = bottled water  2 = tap water  3 = ground  4 = other |
| egg_fd | Frequency of consumption of eggs by participant | 0 = never  1 = rarely (<1 time/month)  2 = sometimes (<=1 time/month but >= 1 time/month)  3 = often (2-3 times / week)  4 = very often (4-6 times / week)  5 = everyday (> = 7 times / week) |
| fast_fd | Frequency of consumption of fast food by participant | 0 = never  1 = rarely (<1 time/month)  2 = sometimes (<=1 time/month but >= 1 time/month)  3 = often (2-3 times / week)  4 = very often (4-6 times / week)  5 = everyday (> = 7 times / week) |
| fat_fd^a^ | Frequency of consumption of fats by participant | 0 = never  1 = rarely (<1 time/month)  2 = sometimes (<=1 time/month but >= 1 time/month)  3 = often (2-3 times / week)  4 = very often (4-6 times / week)  5 = everyday (> = 7 times / week) |
| fish_fd | Frequency of consumption of fish by participant | 0 = never  1 = rarely (<1 time/month)  2 = sometimes (<=1 time/month but >= 1 time/month)  3 = often (2-3 times / week)  4 = very often (4-6 times / week)  5 = everyday (> = 7 times / week) |
| flame_fd12h^a^ | Consumption of grilled food <12h ago | 0 = no  1 = yes |
| flame_fd24h^a^ | Consumption of grilled food <24h ago | 0 = no  1 = yes |
| flame_fd48h^a^ | Consumption of grilled food between 24h and 48h ago | 0 = no  1 = yes |
| flame_fd^a^ | Frequency of consumption of food grilled over an open flame/burning ember by participant | 0 = never  1 = rarely (<1 time/month)  2 = sometimes (<=1 time/month but >= 1 time/month)  3 = often (2-3 times / week)  4 = very often (4-6 times / week)  5 = everyday (> = 7 times / week) |
| fruit_dried_fd^a^ | Frequency of consumption of dried fruits (eg. raisins, apricots, prunes, etc.) | 0 = never  1 = rarely (<1 time/month)  2 = sometimes (<=1 time/month but >= 1 time/month)  3 = often (2-3 times / week)  4 = very often (4-6 times / week)  5 = everyday (> = 7 times / week) |
| fruit_fd^b^ | Frequency of consumption of fruits | 0 = never  1 = rarely (<1 time/month)  2 = sometimes (<=1 time/month but >= 1 time/month)  3 = often (2-3 times / week)  4 = very often (4-6 times / week)  5 = everyday (> = 7 times / week) |
| fruveg_fd^a^ | Frequency of consumption of fruits and vegetables by participant | 0 = never  1 = rarely (<1 time/month)  2 = sometimes (<=1 time/month but >= 1 time/month)  3 = often (2-3 times / week)  4 = very often (4-6 times / week)  5 = everyday (> = 7 times / week) |
| gum_fd^b^ | Frequency of consumption of chewing gum by participant | 0 = never  1 = rarely (<1 time/month)  2 = sometimes (<=1 time/month but >= 1 time/month)  3 = often (2-3 times / week)  4 = very often (4-6 times / week)  5 = everyday (> = 7 times / week) |
| hazel_fd^a^ | Frequency of consumption of hazelnut spread by participant | 0 = never  1 = rarely (<1 time/month)  2 = sometimes (<=1 time/month but >= 1 time/month)  3 = often (2-3 times / week)  4 = very often (4-6 times / week)  5 = everyday (> = 7 times / week) |
| jel_fd^b^ | Frequency of consumption of jelly candies by participant | 0 = never  1 = rarely (<1 time/month)  2 = sometimes (<=1 time/month but >= 1 time/month)  3 = often (2-3 times / week)  4 = very often (4-6 times / week)  5 = everyday (> = 7 times / week) |
| juice_drk^b^ | Frequency of consumption of fruit juice | 0 = never  1 = rarely (<1 time/month)  2 = sometimes (<=1 time/month but >= 1 time/month)  3 = often (2-3 times / week)  4 = very often (4-6 times / week)  5 = everyday (> = 7 times / week) |
| local_fd^a^ | Frequency of consumption of local food by participant | 0 = never  1 = rarely (<1 time/month)  2 = sometimes (<=1 time/month but >= 1 time/month)  3 = often (2-3 times / week)  4 = very often (4-6 times / week)  5 = everyday (> = 7 times / week) |
| meat_fd | Frequency of consumption of meat by participant | 0 = never  1 = rarely (<1 time/month)  2 = sometimes (<=1 time/month but >= 1 time/month)  3 = often (2-3 times / week)  4 = very often (4-6 times / week)  5 = everyday (> = 7 times / week) |
| milk_drk | Frequency of consumption of milk (drinking) | 0 = never  1 = rarely (<1 time/month)  2 = sometimes (<=1 time/month but >= 1 time/month)  3 = often (2-3 times / week)  4 = very often (4-6 times / week)  5 = everyday (> = 7 times / week) |
| organ_meat_fd^a^ | Frequency of consumption of organ meat (offal, liver, kidney, etc.) by participant | 0 = never  1 = rarely (<1 time/month)  2 = sometimes (<=1 time/month but >= 1 time/month)  3 = often (2-3 times / week)  4 = very often (4-6 times / week)  5 = everyday (> = 7 times / week) |
| organic_fd^b^ | Frequency of consumption of organic food by participant | 0 = never  1 = rarely (<1 time/month)  2 = sometimes (<=1 time/month but >= 1 time/month)  3 = often (2-3 times / week)  4 = very often (4-6 times / week)  5 = everyday (> = 7 times / week) |
| plastic_fd^a^ | Frequency of consumption of food from plastic packaging by participant | 0 = never  1 = rarely (<1 time/month)  2 = sometimes (<=1 time/month but >= 1 time/month)  3 = often (2-3 times / week)  4 = very often (4-6 times / week)  5 = everyday (> = 7 times / week) |
| popcorn_fd^a^ | Frequency of consumption of microwave popcorn by participant | 0 = never  1 = rarely (<1 time/month)  2 = sometimes (<=1 time/month but >= 1 time/month)  3 = often (2-3 times / week)  4 = very often (4-6 times / week)  5 = everyday (> = 7 times / week) |
| potato_fried_fd | Frequency of consumption of fried potatoes and/or potato chips | 0 = never  1 = rarely (<1 time/month)  2 = sometimes (<=1 time/month but >= 1 time/month)  3 = often (2-3 times / week)  4 = very often (4-6 times / week)  5 = everyday (> = 7 times / week) |
| poultry_fd^a^ | Frequency of consumption of poultry by participant | 0 = never  1 = rarely (<1 time/month)  2 = sometimes (<=1 time/month but >= 1 time/month)  3 = often (2-3 times / week)  4 = very often (4-6 times / week)  5 = everyday (> = 7 times / week) |
| rice_fd^a^ | Frequency of consumption of rice by participant | 0 = never  1 = rarely (<1 time/month)  2 = sometimes (<=1 time/month but >= 1 time/month)  3 = often (2-3 times / week)  4 = very often (4-6 times / week)  5 = everyday (> = 7 times / week) |
| sea_fd | Frequency of consumption of seafood by participant (includes all food of marine origin, including fish and shellfish, molluscs....) | 0 = never  1 = rarely (<1 time/month)  2 = sometimes (<=1 time/month but >= 1 time/month)  3 = often (2-3 times / week)  4 = very often (4-6 times / week)  5 = everyday (> = 7 times / week) |
| smoke_fd12h^a^ | Consumption of smoked food <12h ago | 0 = no  1 = yes |
| smoke_fd24h^a^ | Consumption of smoked food <24h ago | 0 = no  1 = yes |
| smoke_fd48h^a^ | Consumption of smoked food between 24h and 48h ago | 0 = no  1 = yes |
| smoke_fd^a^ | Frequency of consumption of smoked food by participant | 0 = never  1 = rarely (<1 time/month)  2 = sometimes (<=1 time/month but >= 1 time/month)  3 = often (2-3 times / week)  4 = very often (4-6 times / week)  5 = everyday (> = 7 times / week) |
| source_wtr^a^ | Tap water source at home | 1 = public  2 = private well  3 = both public & private well |
| sugar_drk^b^ | Frequency of consumption of sugar sweetened beverages (eg. soda, non-fresh juice) | 0 = never  1 = rarely (<1 time/month)  2 = sometimes (<=1 time/month but >= 1 time/month)  3 = often (2-3 times / week)  4 = very often (4-6 times / week)  5 = everyday (> = 7 times / week) |
| tea_coffee_drk | Frequency of consumption of tea and coffee | 0 = never  1 = rarely (<1 time/month)  2 = sometimes (<=1 time/month but >= 1 time/month)  3 = often (2-3 times / week)  4 = very often (4-6 times / week)  5 = everyday (> = 7 times / week) |
| veg_fd^b^ | Frequency of consumption of vegetables and vegetable products including fungi | 0 = never  1 = rarely (<1 time/month)  2 = sometimes (<=1 time/month but >= 1 time/month)  3 = often (2-3 times / week)  4 = very often (4-6 times / week)  5 = everyday (> = 7 times / week) |
| vegetarian_fd | Is participant on a vegetarian diet? | 0 = no  1 = yes |
| **Environmental factos** | | |
| cosm_freq^a^ | How often does participant use cosmetics? | 0 = never  1 = rarely (<1 time/month)  2 = sometimes (<=1 time/month but >= 1 time/month)  3 = often (2-3 times / week)  4 = very often (4-6 times / week)  5 = everyday (> = 7 times / week) |
| cosm_rect^a^ | Participant has used cosmetics? | 0 = no  1 = yes |
| biomass_indoor^a^ | Indoor use of coal/biomass burning | 0 = no  1 = yes |
| chem_ind^a^ | Facilities nearby home at time of sampling: industry producing petrochemicals | 0 = no  1 = yes |
| farm_ind^a^ | Facilities nearby home at time of sampling: farmland, orchard, or vinyard | 0 = no  1 = yes |
| traffic^a^ | Density of traffic in the residential area | 0 = no traffic  1 = light traffic  2 = intense traffic |
| waste_inc^a^ | Facilities nearby (within 1 km) home at time of sampling: A waste incineration plant | 0 = no  1 = yes  2 = do not know |

^a^Only used in adults.

^b^Only used in children.

**Table S2.** Distribution of selected exposure variables model by geographical area (North, South and West) in children and adults, separately.

| **Children** | | | |
| --- | --- | --- | --- |
| **Variables** | **North (n = 299)** | **South (n = 300)** | **West (n = 2,559)** |
| **Age, median (IQR) in years** | 10 (9; 11) | 7 (7; 7) | 10 (7; 13) |
| **Body mass index (BMI), median (IQR) in kg/m^2^** | 17 (16; 18) | 16 (15; 18) | 17 (15; 20) |
| **Sex, n (%)** |  |  |  |
| Males | 159 (53) | 150 (50) | 1,272 (50) |
| **Passive smoking exposure at home** |  |  |  |
| Yes | 2 (1) | 24 (8) | 203 (8) |
| **Place of residence, n (%)** |  |  |  |
| Cities | 86 (29) | 229 (76) | 623 (24) |
| Towns/suburbs | 134 (45) | 48 (16) | 1,162 (45) |
| Rural areas | 79 (26) | 23 (8) | 774 (30) |
| **Highest education level of the household, n (%)** |  |  |  |
| Low (ISCED 0 - 2) | - | 26 (9) | 127 (5) |
| Medium (ISCED 3 - 4) | 14 (5) | 139 (47) | 1,000 (40) |
| High (ISCED >= 5) | 273 (95) | 132 (44) | 1,389 (55) |
| **Frequency of food/drinks consumption, n (%)** |  |  |  |
| *Cereals* |  |  |  |
| Never | - | - | 8 (0) |
| Rarely (<1 time/month) | 3 (1) |  | - |
| Sometimes (<=1 time/month but >= 1 time/month) | 8 (3) |  | 987 (39) |
| Often (2-3 times/week) | 79 (26) |  | 748 (29) |
| Very often (4-6 times/week) | 60 (20) |  | 294 (11) |
| Everyday (>= 7 times / week) | 91 (30) |  | 452 (18) |
| Missing | 58 (19) |  | 79 (3) |
|  |  |  |  |
| *Fried potatoes and/or potato chips* |  |  |  |
| Never | - | - | 64 (3) |
| Rarely (<1 time/month) |  |  | 33 (1) |
| Sometimes (<=1 time/month but >= 1 time/month) |  |  | 1,761 (69) |
| Often (2-3 times/week) |  |  | 495 (19) |
| Very often (4-6 times/week) |  |  | 90 (4) |
| Everyday (>= 7 times / week) |  |  | 29 (1) |
| Missing |  |  | 87 (3) |
| *Bread* |  |  |  |
| Never | - | - | 8 (0) |
| Rarely (<1 time/month) | 3 (1) |  | 1 (0) |
| Sometimes (<=1 time/month but >= 1 time/month) | 11 (4) |  | 233 (9) |
| Often (2-3 times/week) | 22 (7) |  | 547 (21) |
| Very often (4-6 times/week) | 52 (17) |  | 385 (15) |
| Everyday (>= 7 times / week) | 151 (51) |  | 1,309 (51) |
| Missing | 60 (20) |  | 76 (3) |
| *Fast food* |  |  |  |
| Never | - | - | 199 (8) |
| Rarely (<1 time/month) | 173 (58) |  | - |
| Sometimes (<=1 time/month but >= 1 time/month) | 66 (22) |  | 1,909 (75) |
| Often (2-3 times/week) | - |  | 387 (15) |
| Very often (4-6 times/week) | - |  | 18 (1) |
| Everyday (>= 7 times / week) | - |  | 26 (1) |
| Missing | 60 (20) |  | 20 (1) |
| *Tea & coffee* |  |  |  |
| Never | - | - | 597 (23) |
| Rarely (<1 time/month) | 205 (69) |  | 27 (1) |
| Sometimes (<=1 time/month but >= 1 time/month) | 21 (7) |  | 1,020 (40) |
| Often (2-3 times/week) | - |  | 236 (9) |
| Very often (4-6 times/week) | 6 (2) |  | 104 (4) |
| Everyday (>= 7 times / week) | 2 (1) |  | 503 (20) |
| Missing | 65 (22) |  | 72 (3) |
| **Adults** | | | |
| **Variables** | **North (n = 203)** | **South (n = 411)** | **West (n = 684)** |
| **Age, median (IQR) in years** | 31 (26; 35) | 35 (31; 37) | 31 (25; 36) |
| **BMI, median (IQR) in kg/m^2^** | 25 (23; 29) | 24 (21; 27) | 23 (21; 26) |
| **Sex, n (%)** |  |  |  |
| Males | 89 (44) | 123 (30) | 318 (46) |
| **Place of residence, n (%)** |  |  |  |
| Cities | 153 (77) | 166 (41) | 356 (52) |
| Towns/suburbs | 23 (12) | 133 (32) | 171 (25) |
| Rural areas | 23 (12) | 110 (27) | 157 (23) |
| **Highest education level of the subject, n (%)** |  |  |  |
| Low (ISCED 0 - 2) | 12 (6) | 67 (16) | 16 (2) |
| Medium (ISCED 3 - 4) | 60 (30) | 131 (32) | 155 (23) |
| High (ISCED >= 5) | 128 (64) | 213 (52) | 513 (75) |
| **Frequency of food/drinks consumption, n (%)** |  |  |  |
| *Alcoholic beverages* |  |  |  |
| Never | 46 (23) | 110 (27) | 49 (7) |
| Occasionally (<1/week) | 17 (8) | 128 (31) | 121 (18) |
| Frequently (>=1 week) | 117 (58) | 148 (36) | 320 (28) |
| Missing | 23 (11) | 25 (6) | 194 (28) |
| *Fried potatoes and/or potato chips* |  |  |  |
| Never | 57 (28) | 16 (4) | 54 (8) |
| Rarely (<1 time/month) | 48 (24) | 74 (18) | 57 (8) |
| Sometimes (<=1 time/month but >= 1 time/month) | 13 (6) | 144 (35) | 283 (41) |
| Often (2-3 times/week) | 41 (20) | 87 (21) | 87 (13) |
| Very often (4-6 times/week) | 11 (5) | 55 (13) | 7 (1) |
| Everyday (>= 7 times / week) | 9 (4) | 11 (3) | 1 (0) |
| Missing | 24 (12) | 24 (6) | 195 (28) |
| *Coffee* |  |  |  |
| Never | 46 (23) | 44 (11) | 87 (13) |
| Rarely (<1 time/month) | 2 (1) | 4 (1) | 20 (3) |
| Sometimes (<=1 time/month but >= 1 time/month) | - | 4 (1) | 24 (3) |
| Often (2-3 times/week) | 15 (7) | 10 (2) | 25 (4) |
| Very often (4-6 times/week) | 11 (5) | 2 (0) | 37 (5) |
| Everyday (>= 7 times / week) | 106 (52) | 47 (11) | 190 (28) |
| Missing | 23 (11) | 100 (73) | 301 (44) |
| *Fruits and vegetables* |  |  |  |
| Never | - | - | 5 (1) |
| Rarely (<1 time/month) | - | - | 6 (1) |
| Sometimes (<=1 time/month but >= 1 time/month) | 8 (4) | 1 (0) | 31 (4) |
| Often (2-3 times/week) | 45 (22) | 16 (4) | 75 (11) |
| Very often (4-6 times/week) | 34 (17) | 30 (7) | 128 (19) |
| Everyday (>= 7 times / week) | 90 (44) | 340 (83) | 425 (62) |
| Missing | 26 (13) | 24 (6) | 14 (2) |
| *Bread* |  |  |  |
| Never |  | 1 (0) | 6 (1) |
| Rarely (<1 time/month) |  | 5 (1) | 8 (1) |
| Sometimes (<=1 time/month but >= 1 time/month) |  | 6 (1) | 51 (7) |
| Often (2-3 times/week) | - | 24 (6) | 154 (22) |
| Very often (4-6 times/week) |  | 59 (14) | 143 (21) |
| Everyday (>= 7 times / week) |  | 292 (71) | 308 (45) |
| Missing |  | 24 (6) | 14 (2) |
| *Cereals* |  |  |  |
| Never | 20 (10) | 60 (15) | 1 (0) |
| Rarely (<1 time/month) | 8 (4) | 5 (1) | - |
| Sometimes (<=1 time/month but >= 1 time/month) | 25 (12) | 4 (1) | - |
| Often (2-3 times/week) | 56 (28) | 17 (4) | 6 (1) |
| Very often (4-6 times/week) | 37 (18) | 3 (1) | 29 (4) |
| Everyday (>= 7 times / week) | 32 (16) | 22 (5) | 168 (25) |
| Missing | 25 (12) | 300 (73) | 480 (70) |
| *Cookies, biscuits and cakes* |  |  |  |
| Never | 16 (8) | 12 (3) | 151 (22) |
| Rarely (<1 time/month) | 44 (22) | 86 (21) | 9 (1) |
| Sometimes (<=1 time/month but >= 1 time/month) | 4 (2) | 101 (25) | 153 (22) |
| Often (2-3 times/week) | 95 (47) | 120 (29) | 98 (14) |
| Very often (4-6 times/week) | 15 (7) | 15 (4) | 46 (7) |
| Everyday (>= 7 times / week) | 6 (3) | 53 (13) | 33 (5) |
| Missing | 23 (11) | 24 (6) | 194 (28) |

n: total number of subjects included in the analysis; ISCED: International Standard Classification of Education; - : information not available in the dataset; IQR: Interquartile range.

**Table S3.** Pooled crude and multivariable-adjusted median differences in AAMA and GAMA urinary levels (beta coefficients with 95% CI) in relation to European geographical regions (North vs South and West). Results are presented for the whole sample of children/adolescents and adults, respectively, including passive and active smokers.

| **Population group** | **Model** | **AA urinary biomarkers** | **European geographical region** | | | |
| --- | --- | --- | --- | --- | --- | --- |
|  |  |  | **North** | **South** | **West** | **N** |
|  |  |  | **Reference** | **β (95% CI)**  **(µg/g creatinine)** | **β (95% CI)**  **(µg/g creatinine)** |  |
| Children/Adolescents | Crude | AAMA | Ref. | 27.3 | 8.5 | 3,157 |
|  |  |  |  | (20.3, 34.3) | (3.3, 13.7) |  |
|  |  | GAMA | - | 22.4 | 3.9 | 3,157 |
|  |  |  |  | (21.1, 23.7) | (2.9, 4.9) |  |
|  | Multivariable-adjusted^a^ | AAMA | - | 25.5 | 27.6 | 2,996 |
|  |  |  |  | (-8.7, 59.8) | (7.5, 47.6) |  |
|  |  | GAMA | - | 25.3 | 7.3 | 2,996 |
|  |  |  |  | (18.0, 32.6) | (3.0, 11.5) |  |
| Adults | Crude | AAMA | - | 23.0 | 0.8 | 1,297 |
|  |  |  |  | (15.0, 31.0) | (-6.6, 8.3) |  |
|  |  | GAMA | - | 14.9 | 0.25 | 1,281 |
|  |  |  |  | (13.9, 16.0) | (-0.71, 1.20) |  |
|  | Multivariable-adjusted^b^ | AAMA | - | 22.2 | -20.4 | 1,220 |
|  |  |  |  | (9.8, 34.6) | (-35.8, -5.1) |  |
|  |  | GAMA | - | 6.6 | -3.0 | 1,206 |
|  |  |  |  | (4.7, 8.6) | (-5.4, -0.6) |  |

^a^Model including the following variables: sex, age, passive smoking, sampling year, place of residence (cities, town and rural areas), highest educational level, body mass index (BMI), physical activity, and frequency of consumption of seafood, fish, meat, cereals, fried potatoes, bread, fast food, sugar drinks, and tea/coffee. Smoking not included in the model due to the presence of collinearity.

^b^Model including the following variables: smoking, sex, sampling year, age, place of residence (cities, town and rural areas), educational level, physical activity, BMI, frequency of consumption of seafood, fried potatoes, fruits/vegetables, coffee, and alcohol.

**Figure S1.** Pooled multivariable-adjusted^1^ median differences in AAMA and GAMA urinary levels (beta coefficients and 95% CI in µg/g creatinine) in relation to non-dietary and dietary determinants. Results are presented for the whole sample of children/adolescents (a) and adults (b), respectively^2^, including passive and active smokers.

Educational level classified according to ISCED: international standard classification for education (for children referred to the maximum reported in the household); BMI: body mass index

^1^In children/adolescents, the variable "smoking" was not included in the model due to collinearity, and adjustments were made for variables shown in Figure (a) plus participating study, sampling year, physical activity, consumption of seafood, fish, meat and sugar drinks; in adults, adjustments were made for variables shown in Figure (b) plus participating study, sampling year, physical activity, vegetarian diet, and consumption of seafood, fish, meat and milk.

^2^For a better readability of the results, raw data of the following variables were multiplied by a factor of 25: cereals, bread, fast food and tea/coffee in children/adolescents; and age, BMI, cereals, fruit/vegetables and coffee in adults.

*Age, BMI and dietary variables (frequency of consumption) treated as continuous (unit increase).

**SI-1. Analytical methods for determination of AAMA and GAMA in urine of the HBM4EU Aligned and participating studies included in the present research.**

Six different laboratories, including the Institute and Outpatient Clinic of Occupational, Social and Environmental Medicine (IPASUM, Friedrich-Alexander-Universität Erlangen-Nürnberg, Germany), Analytisch-Biologisches Forschungslabor (ABF, Germany), Lausanne Forensic Toxicology and Chemistry Unit (University Center of Legal Medicine, Switzerland), Institute of Biomonitoring (Currenta GmbH&Co.OHG, SEL-SER-GS, Germany), Laboratory of Environmental and Industrial Toxicology (University of Milan, Italy), and FISABIO-Food Safety Research Area (Valencia, Spain) performed the urinary analysis of both biomarkers (AAMA and GAMA) in the HBM4EU Aligned and participating studies included in the present research. Each laboratory applied a different methodology for urinary analysis. In brief, the volume of sample ranged from 0.1 to 2 ml, and urine treatment consisted of dilution (n = 4) or evaporation and reconstitution in methanol (n = 2) [1], [2]. Liquid chromatography coupled to tandem mass spectrometry (LC-MS/MS) was used for AAMA and GAMA determination in all laboratories, with limits of quantification (LOQs) between 0.25 and 5 ng/ml. The quality of the analytical results was assured either by successful qualification in the HBM4EU quality assurance/quality control (QA/QC) scheme [3], with generated data labelled as (A) “Biomarker data quality assured by HBM4EU QA/QC programme” or (B) “Biomarker data generated before HBM4EU QA/QC programme but deemed comparable by HBM4EU Quality Assurance Unit (QAU)”, or by participation in External Quality Assessment Schemes (EQUAS) intercomparison programs (e.g., G-EQUAS) different from the HBM4EU QA/QC scheme and following a high-quality QA/QC system, including the use of in-house QCs and internal standards, which was labelled as (C) “Biomarker data generated before HBM4EU QA/QC programme but comparability not guaranteed by HBM4EU QAU”.

**References**

[1] HBM4EU, “Additional deliverable 9.9: Compilation of the analytical methods used by the HBM4EU laboratories for the substances on the 1st and 2nd prioritisation list (version 1.0),” 2022.

[2] S. F. Fernández, O. Pardo, C. Coscollà, and V. Yusà, “Exposure assessment of Spanish lactating mothers to acrylamide via human biomonitoring,” *Environ Res*, vol. 203, no. June 2021, 2022, doi: 10.1016/j.envres.2021.111832.

[3] HBM4EU, “D9.4 - The Quality Assurance / Quality Control Scheme in the HBM4EU project,” 2017.
